# Pulmonary Balloon Valvuloplasty during Pregnancy

**DOI:** 10.1155/2012/353168

**Published:** 2012-11-11

**Authors:** Mustafa Oylumlu, Kazim Aykent, Hatice Ender Soydinc, Muhammed Oylumlu, Faruk Ertas, Hasan Orhan Ozer, Ibrahim Sari

**Affiliations:** ^1^Department of Cardiology, Faculty of Medicine, Dicle University, 21280 Diyarbakir, Turkey; ^2^Department of Cardiology, Faculty of Medicine, Gaziantep University, 27310 Gaziantep, Turkey; ^3^Department of Obstetrics and Gynecology, Faculty of Medicine, Dicle University, 21280 Diyarbakir, Turkey

## Abstract

Women with valvular heart disease have an increased risk of adverse outcomes in pregnancy; however, with appropriate evaluation and treatment, most women can successfully bear healthy children. During pregnancy, pulmonary stenosis is generally well tolerated in the absence of other haemodynamically significant lesions. We present a case of a multiparous woman,who is pregnant with her sixth child, with a severe pulmonary stenosis. She presented with exertional chest pain and dyspnea. She was managed successfully with balloon valvuloplasty.

## 1. Introduction 

 Pregnancy induces changes in the cardiovascular system to meet the increased metabolic demands of the mother and fetus. Plasma volume reaches a maximum of 40% above baseline at 24 weeks of gestation. A 30–50% increase in cardiac output occurs in normal pregnancy. Heart rate starts to rise at 20 weeks and increases until 32 weeks [1]. This increase in hemodynamic load can present a risk for the development of cardiovascular complications. Maternal heart disease complicates approximately 4% of all pregnancies [2]. However, it accounts for 10% to 25% of maternal mortality [3]. Pulmonary valve stenosis is common problem in pregnancy because of patients can reach adulthood without symptoms, even if the gradient through the stenotic valve is high. During pregnancy, right ventricular obstruction tends to be very well tolerated despite the gestational volume overload imposed on an already pressure-loaded right ventricle. Since the first percutaneous balloon pulmonary valvuloplasty, reported by Kan et al. in 1982, this procedure has become the main treatment for pulmonary stenosis [4]. In the literature, pulmonic balloon valvuloplasty during pregnancy is very rare [5]. We present a pregnant woman with severe pulmonary valve stenosis who was treated successfully with balloon valvuloplasty.

## 2. Case

 A 32-year-old Caucasian female at 28 weeks of gestation presented to our clinics with mild exertional chest pain, coupled at times with mild dyspnea, for the past month. She described her pain as centrally located, heavy, crushing, without any radiation, and not of a musculoskeletal nature. Her pain was worse on exertion and alleviated by rest. She had no associated symptoms such as palpitations, syncope, dizziness, orthopnea, or paroxysmal nocturnal dyspnea and had no significant risk factors such as diabetes mellitus, hypertension, or dyslipidemia. She is a nonsmoker and has a New York Heart Association (NYHA) Functional Classification of Class II. Her past medical history is significant for severe pulmonary stenosis diagnosed in 2003, which she is continuing to be monitored for. She is currently not on any medications and has an obstetric history, not inclusive of her current pregnancy, and of G4P5 with normal vaginal delivery and no complications. Her last delivery was that of twins in 2002 and all her children are well and thriving. She has no significant family history for cardiac conditions.

On physical examination, the patient was alert, oriented, and cooperative. Her vitals were as follows: blood pressure of 100/60 mmHg, heart rate of 96 beats per minute with a regularly regular pulse, respiratory rate of 14 breaths per minute, SpO_2_ of 99% on room air, and she had a body mass index of 38.95 kg/m^2^. She had no peripheral stigmata of cardiac disease, capillary refill was <2 seconds, and she had no conjunctival pallor or central cyanosis. Her jugular venous pressure was not raised, her apex beat was not felt, and no heaves or thrills were present on palpation. Her heart sounds were dual with an early ejection systolic murmur of 3+/6+ heard loudest over the left upper sternal border. Her chest was clear with no added breath sounds with equal entry of air bilaterally. She displayed no other signs indicative of cardiac failure. 

**Figure 1:**
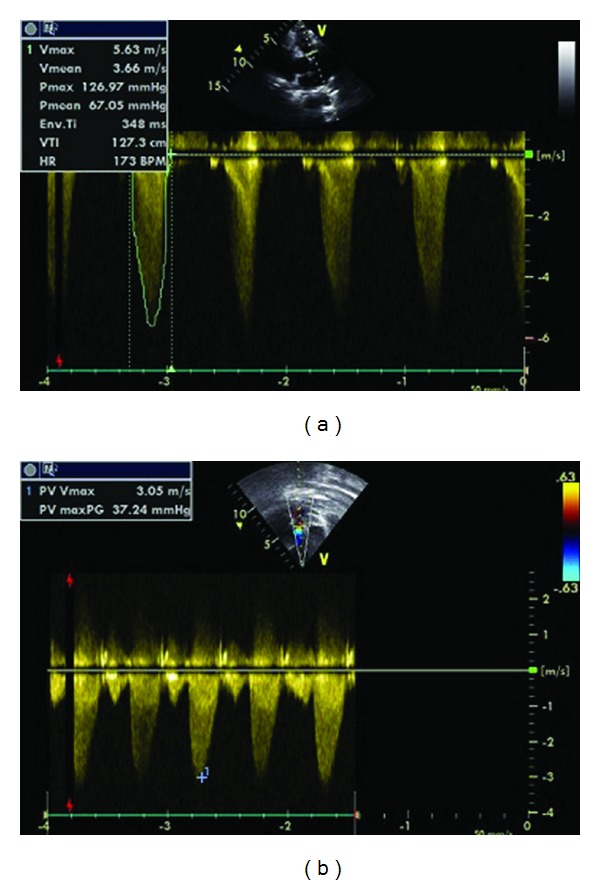
The figure shows pulmonary valve stenosis with a maximum gradient of 126 mmHg before pulmonary balloon valvuloplasty (a) and mild pulmonary valve stenosis with a maximum gradient of 37 mmHg after pulmonary balloon valvuloplasty (b).

An electrocardiogram revealed the patient to be in sinus rhythm with right axis deviation, right bundle branch block, right ventricular hypertrophy, and nonspecific ST-T wave changes. Results from blood tests were unremarkable with no elevation in the cardiac enzymes. Chest X Ray showed an increased cardiac-to-thoracic ratio with right ventricular enlargement and clear costophrenic recesses. Echocardiogram showed pulmonary valve stenosis with a maximum gradient of 126 mmHg, Figure 1(a). The patient also had a widening of the chambers in the right side of the heart as well as right ventricular hypertrophy and dilatation. A moderate tricuspid valve insufficiency was also noted. The left ventricular function was normal at an ejection fraction of 65%. Pulmonary valvuloplasty was performed and was successful. On postprocedural echocardiography the pulmonary gradient fell to 37 mmHg and no complications from the procedure developed thereafter, Figure 1(b). 

## 3. Discussion

0.2–4% of all pregnancies in Western industrialized countries are complicated by cardiovascular diseases [2]. Treatment of congenital heart disease has improved, resulting in an increased number of women with heart disease reaching childbearing age [6]. In the Western world, congenital heart disease is the most frequent cardiovascular disease present during pregnancy (75–82%). However, in non-Western world, rheumatic valvular disease is the most common cardiovascular disease present during pregnancy (56–89%) [7].

Almost all cases of valvular pulmonic stenosis are congenital in origin. Pulmonary stenosis (PS) accounts for 10% to 12% of congenital heart disease in adults and the probability of survival to child bearing age is high [8]. Isolated PS is rarely a significant impediment to a successful pregnancy [9]. Mild-to-moderate PS is associated with little or no maternal risk [10]. Severe PS can be well tolerated during pregnancy. However, severe PS may be associated with increased risk during labor, delivery, and the puerperium. Hameed et al. demonstrated no significant impact of PS with isolated and normal right ventricular function on maternal and fetal wellbeing regardless of the severity [11]. Most patients demonstrated clinical stability without a significant impact of pregnancy on functional status. Favourable maternal outcome in patients with PS is confirmed by other studies. A review summarizing data in approximately 100 patients with PS published in 6 different studies reported no cases of arrhythmias, heart failure, and endocarditis [12]. However, Drenten et al. reported 108 pregnancies in 51 patients with isolated pulmonary valvular stenosis observed a higher than expected number of serious pregnancy/obstetric (e.g., hypertension-related disorders, miscarriages, thromboembolic complications, and premature rupture of membranes) and neonatal (e.g., premature delivery and offspring mortality) complications [13]. A recent review demonstrated [9] that there were no cardiac complications (arrhythmia, heart failure, or other cardiovascular events) in over 100 pregnancies. With respect to the fetus, premature delivery occurred in 16 of 110 pregnancies (14.5%), fetal mortality in 1 of 123 pregnancies (0.8%), perinatal mortality in 5 of 123 pregnancies (4.1%), and recurrent congenital heart disease (of any type) in offspring in 3 of 104 pregnancies (2.8%). 

 American Heart Association/American College of Cardiology practice guidelines have recommended balloon valvuloplasty in asymptomatic nonpregnant patients with PS when the peak gradient across the pulmonic valve is greater than 40 mm Hg [10]. The performance of balloon valvuloplasty during pregnancy may impact unfavorably on fetal wellbeing secondary to the use of ionizing radiation and the potential for hemodynamic instability during the procedure. The effects of radiation on the fetus depend on the radiation dose and the gestational age at which exposure occurs. If possible, procedures should be delayed until at least the completion of the period of major organogenesis (12 weeks after menses). There is no evidence of an increased fetal risk of congenital malformations, intellectual disability, growth restriction, or pregnancy loss at doses of radiation to the pregnant woman of 50 mGy [1]. Most medical procedures do not expose the fetus to such high levels of radiation. For the majority of diagnostic medical procedures, radiation dose to the fetus is less than 1 mGy. Percutaneous balloon valvotomy performed during pregnancy with severe PS has reduced the peripartum risk [5]. Thus, we perform balloon valvuloplasty because of her functional status NYHA 3-4 and there is no problem for radiation exposure.

Our patient presented with the later and despite all the predicted unfavourable outcomes present for each of her conditions separately, she still managed to remain asymptomatic during pregnancy and birth with her prior 5 children. The question of further cardiac complications during her next pregnancy remains for discussion and further research. Often these studies aim to stratify risk of maternal cardiac complications and fetal complications prior to a woman's first pregnancy; however, there is little known in the way of cardiac complication and fetal complication risk stratification for multiparous women with congenital cardiac conditions.
